# Integrating Narrative Medicine Through Story-Telling: A Feasibility Study in a Community Medicine Curriculum for Undergraduate and Postgraduate Students

**DOI:** 10.7759/cureus.41851

**Published:** 2023-07-13

**Authors:** Madhavi Bhargava, Poonam R Naik, Pavithra Hegde, Nagendra Navya, Malavika Sachith, Sathiamoorthy Vineetha

**Affiliations:** 1 Community Medicine, Yenepoya Medical College, Mangalore, IND

**Keywords:** communication skills, social determinants, medical education, story-telling, narrative medicine

## Abstract

Introduction

The routine curriculum of community medicine includes clinico-social case-taking with a focus on the physical, biological, and psychosocial determinants of health. There is an opportunity to integrate narrative medicine with this for undergraduate and postgraduate medical students using story-telling. The objective of the current study was to assess its feasibility, challenges, and opportunities.

Methods

We conducted a need assessment cross-sectional survey of the teaching faculty of community medicine across India using Google Forms. Considering an 80% positive response in a pilot within the department, a relative error of 10%, and a 20% non-response rate, the sample size was 120. The questionnaire included closed-ended questions with a Likert scale that dealt with affective, cognitive, and communication domains and open-ended questions for insights into opportunities and challenges. The results of the former were expressed as descriptive statistics, in frequencies and proportions. Open-ended questions were summarized to guide the refinement of further implementation.

Results

Of the 120 participants, 92 (77%) quoted low/medium empathy quotient in students, and 107 (89.2%) felt that the listening skills of students can improve with the introduction of story-telling. A hundred and twelve (93.4%) participants felt that their history-taking skills can improve with story-telling, and all agreed that the language of medicine can be improved. One hundred nine (90.8%) felt that it will lead to better student-patient interaction. Opportunities included a better understanding of social determinants, patient-/family-centered care, improved communication skills, and better mental health. The key challenges included time, motivation, the need for training/capacity building, and streamlining of assessment metrics.

Conclusion

We conclude that story-telling may help medical students investigate various social determinants of health, disease, and lived environments that create vulnerabilities.

## Introduction

We are in an era where healthcare is specialty, super-specialty, and technology-driven, with impressive new advances occurring in medical science. However, this has not reduced the role of humanities and narrative medicine in the clinical care environment, an environment filled with fear, anxiety, pain, and suffering. On the contrary, it is even more relevant in current times. Sharing experiences that are narrated by the patient and their caregiver can overcome the harmful barriers in a stressful environment and improve the coping mechanisms during a vulnerable stage of illness in a person’s life [1]. Narrative medicine as a part of literature and medicine was a term coined by Rita Charon and is defined as “practicing medicine with narrative competence [2].” It is also defined as “medicine practiced with narrative skills of recognizing, absorbing, interpreting, and being moved by the stories of illness [3].” Narrative medicine can provide a respectful environment that is empathic and wholesome and is capable of nourishing medical care positively. To this end, we need to recognize situations between the doctor and the patient within the medical fraternity, between the doctor and the society, to connect to oneself with enriching narratives [2]. This can help understand the illness, patient's and caregiver’s sufferings, disability (momentary or lifelong), personhood, and therapy (medical as well as non-medical) much better and promote teamwork and cohesion within the healthcare team [4]. It has the potential to build skills and competencies in communication, collaboration, empathy, and reflection that are otherwise not routinely addressed by the traditional medical training approach. These skills are essential buffers against burnout and "compassion fatigue" that can creep into clinical care work [5,6].

Story-telling is a type of narrative medicine that can integrate our understanding of symptoms, signs, laboratory findings, and diagnosis of a disease in a more holistic way with patients and their families at the center of it all [4]. What may be a routine for healthcare providers, an illness is a phase that is critical for the patient and the family in their life course. Being able to see this from a human angle is a skill that can mold medical students, the future doctors, into thoughtful, capable, and empathetic individuals. A good story-telling session can create the ability of the story-teller and the listener in engaging with patients, communities, and their peers in an effective way. It can provide a good mix of what is known as the art and science of medicine.

There have been several initiatives with systematic implementation and documentation, at times for better communication of science to the general public or for teaching ethics, professionalism, better reading, and sharing culture in many countries [4,7-9]. Narrative medicine is yet to gain momentum in Indian medical education.

Thus, we at the Department of Community Medicine in a tertiary teaching hospital in South India planned innovation in undergraduate and postgraduate students' curricula to introduce the activity to learn to tell, listen, and relate to stories of patients and families they encounter during their clinical postings. The objective of this study was to conduct a need assessment survey among the fraternity of community medicine teaching faculty across India and to identify the opportunities and challenges in introducing this innovation.

## Materials and methods

Clinico-social case-taking in community medicine

During the routine curriculum in community medicine, a clinico-social case-taking and discussion touch upon various aspects of the determinants of health. These are physical, biological, and psychosocial environments, living and housing conditions, economic distress and medical debts, water, and sanitation, issues of access to healthcare, the influence of education on health and disease, cultural practices, equity, and justice, to name a few.

Story-telling: a step further in routine clinico-social case-taking

A clinico-social case discussion provides a fertile ground to see the health problem in a wholesome context rather than in isolation as a medical condition. It helps look into various social determinants of health, disease, and lived environment that create vulnerabilities. It may even contribute in a direct or indirect manner toward the protection of not only the patient but also the family and the community.

Proposed modes of story-telling

It is a combination of written and oral narration regarding the etiology of an illness or selected diseases and understanding the related social determinants. It provides fertile ground to study the interplay between the disease or health condition, the family, the symptomatology as perceived by the patient and the relatives, and their coping mechanism in terms of grief, pain, financial crisis, or disability. It might even be a humorous or tricky situation in a healthcare setting that happens so often and can lighten the tense moments. A small percentage of routine clinico-social case discussions related to tuberculosis, antenatal mothers, diarrheal diseases, non-communicable diseases, and disability (social determinants relevant to these clinico-social cases) can be in the form of story-telling.

Learning objectives and teaching-learning domains covered

The specific objectives are to enhance reflective thinking, communication skills, creative language, and writing skills and inculcate the quality of empathy. The activity intends to cover the affective, cognitive, and communication domains. Students are likely to learn the skills of responding appropriately to a health or a medical situation, and the resultant discussion that follows can provide alternative emotional responses. This improves the understanding of how different people approach the same situation differently and, therefore, appreciate diversity. Telling and listening to stories can enhance the narrative and communication skills that can start as mere observations but lead to better articulation and internalization of good communication that may gradually come naturally. The story-teller and the listener may be at a different stage in each of these skills and can navigate these at their own pace.

The need assessment survey among the community medicine fraternity

We conducted a need assessment using a cross-sectional survey of the teaching faculty of community medicine across India to understand the opportunities and challenges in story-telling for better implementation. We used an online data collection process (Google Forms) using professional email groups and personal contacts. The questionnaire included closed-ended questions with a 5-point Likert scale and some open-ended questions. The closed-ended questions dealt with affective, cognitive, and communication domains, and the open-ended questions were for insights into opportunities and challenges. The questions and components of the need assessment survey tool were validated by three subject experts who did not participate in the feasibility study. The results of the closed-ended questions were expressed as descriptive statistics, in frequencies and proportions. Open-ended questions were summarized to guide the refinement of further implementation.

Sample size and sampling technique of the need-assessment component

An initial discussion among the department faculty was conducted, and we found that 80% of the teaching faculty indicated a positive response toward feasibility and need. The institutional board of studies (that includes subject experts from other medical colleges) also gave a positive response to the proposed activity. Considering this as a pilot and considering an 80% prevalence of positive response, with a relative error of 10% and a 20% non-response rate, the sample size was 120. We used convenience sampling through professional/medical education email groups. Data collection for need assessment was done between 1st August 2021 and 15th October 2021. The discussions and activities in the department guided the need assessment survey and the scope of dissemination among the peers. Ethics clearance was obtained from the institutional ethics committee for the need assessment component, and a due informed consent process was followed. All information, views, and opinions were anonymous and confidential.

## Results

Of the 120 participants that were surveyed, 92 (77%) felt that the empathy quotient of the present-day medical students is low or medium, and 107 (89.2%) felt that the listening skills of students will improve with the introduction of story-telling. A hundred and twelve (93.4%) participants felt that the history-taking skills of students can improve with story-telling, and all agreed that the language of medicine can be made in a more story-like context. One hundred nine (90.8%) felt that it will lead to better student-patient interaction, and on being asked if the increased time that the student will spend occasionally in the process of creating a story will improve patient satisfaction, 99 (82.5%) agreed; however, seven (5.8%) disagreed. This is depicted in Table 1.

**Table 1:** Learning domains that can be addressed by story-telling sessions among undergraduate and postgraduate medical students *No participant disagreed Survey results from faculty across India

Domain	Questions		Frequency	Percentage
Affective Domain	How would you rate the empathy quotient among medical students on a scale of high to low?	High	28	23.3
		Medium	64	53.4
		Low	28	23.3
	Listening skills will improve among students using story-telling.	Agree	107	89.2
		Neutral	9	7.5
		Disagree	4	3.3
Cognitive Domain	Involvement of story-telling in the curriculum is likely to improve the history-taking skill among medical students.	Agree	112	93.4
		Neutral	7	5.8
		Disagree	1	0.8
Affective and Cognitive Domain	Language of medicine can be made in a story-like context to help the students visualize and learn*.	Highly agree	49	40.8
		Agree	65	54.2
		Neutral	6	5.0
Affective and Communication Domain	Story-telling can enable students to interact with the patients rather than just going through the file.	Agree	109	90.8
		Neutral	9	7.5
		Disagree	2	1.7
Communication Domain	Increased student patient time for story-telling will lead to better patient satisfaction.	Agree	99	82.5
		Neutral	14	11.7
		Disagree	7	5.8

Some of the opportunities pointed out by the participants included a better understanding of the social determinants of health. Moreover, the symptoms that are most important for the patient and their families (that may not be the same as medically important symptoms) can guide the management plan, set appropriate priorities, and improve the acceptability of the treatment among the patients. Story-telling is likely to give an opportunity for the students to understand various challenges and coping mechanisms of the patients and their families and also how communities bear the cost of care. It has the potential to improve communication skills as far as breaking bad news and long-term palliative care is concerned. It can contribute to improving societal mental health and fabric due to better communication.

Among the challenges, the most important challenge expressed was that of time. The new curriculum that is being implemented in India has reduced hours allocated for community medicine as compared to earlier. This needs to be kept in mind while implementing the story-telling sessions in the curriculum. There may be challenges in communicating the importance of story-telling and may require additional teacher training and capacity-building activities to generate interest and motivation. Moreover, any activity that is not assessed in medical education may not be taken seriously; thus, a need to design an assessment technique was expressed. An activity like this cannot be standardized and may not always proceed as planned. As a result, it may require a lot of patience from all the stakeholders and an environment conducive to mutual learning.

Participants also came up with suggestions alongside the challenges for better implementation. Making story-telling an elective, designing implementable training modules, dedicating a specific number of hours, and identification of suitable topics were some of them. An important suggestion was to start with postgraduate students, and the lessons learned from there can be useful for undergraduate story-telling. Small group teaching methods and training from social scientists were other important suggestions. A suggestion to start it on a smaller scale like a literary event or a cultural activity that may help generate ideas and refine the concept was also a very useful one.

The opportunities, challenges, and suggestions are summarized in Table 2.

**Table 2:** Role of story-telling in various aspects of the healthcare delivery process: opportunities, challenges, and suggestions to overcome them

Opportunities	Challenges	Suggestions
Addresses the understanding of social determinants of health and disease	Time constraints; reduced hours for community medicine as a subject in the new curriculum	Can be introduced as an elective
Understanding symptomatology of disease as perceived and prioritized by the patient, their relatives, and caregivers; understanding the difference between "disease and illness"	Communicating the concept, its importance, and its need and inculcating it in the existing system may require extra effort	Make implementable training modules
Symptomatology and problems as perceived by the relatives and caregivers and create management partnerships	Teacher training, capacity building, interest generation, motivation	Defined hours allocated to this activity
Can guide the management plan and improve the faith of the patients, and treatment acceptability	Resistance from the teaching fraternity	Identify suitable topics within the subject to make them useful
Understanding the difficulties and the coping mechanisms of the patient and the family, including the cost of care	Any activity that is not assessed may not be taken seriously by the students: how is it going to be assessed?	Start with postgraduates and later introduce to undergraduate students; suitable for small group discussions. Start on a smaller scale in the form of literary/cultural events. It will help generate ideas, and innovation and improve the concept
A good tool for improving mental health	Cannot be standardized	Training from social scientists with semi-structured templates that highlight the bi-directionality of illness and social factors
Will help learn skills required to break bad news, explain palliative care	Patience from all stakeholders: patients, students, and teachers. May not always go as planned	Timely feedback from all stakeholders

## Discussion

The need assessment survey, background work, and suggestions that we received from community medicine teaching faculty across the country have helped in refining our implementation strategy. There are no pre-existing modules to cultivate this skill, either as listeners or story-tellers, but the fact remains that it is crucial not just in community medicine but also in any medical discipline [1]. It has the potential to improve the feeling of kinship not just with the patients and their families and create a humane discourse in the medical field with each stakeholder as a partner in it [2]. While narrative medicine is being used extensively by the medical humanities and ethics fraternity, it has not been integrated in a major way into routine medical education. At best, narratives have been used by few medical professionals or teachers to narrate how they navigate challenging situations. This can be very influential to young students and can even shape the way they look at mundane medical situations every day.

An important barrier is with the teachers who feel that they may not be equipped with the skills of story-telling. Just as skills of good history-taking and case presentations, writing interesting case reports, and the skill of public speaking come with practice, the same is the case with weaving stories around a situation in healthcare.

Ventres and Gross suggest implementation strategies where the teachers “instead of pushing facts, pull the audience/students by their narrative skills and stories” and help people see what they see [1]. They suggest a process-oriented approach and a question-oriented approach. The process-oriented approach identifies themes at all stages of an initiative to tell a story, right from recognizing them and reflecting on them to identifying the characters, presenting in an engaging way, and being responsive to feedback from the audience. The question-based approach includes asking why, what, how, and what else in a situation, followed by answering some basic questions as to what the story-teller (and the listener) learned, did that change the way they saw things earlier, and if possible, describe these changes. They even provide resources to learn and publish these stories. An important barrier of time and logistics is well recognized in past studies. This was a common barrier noted by Fox and Hauser in their qualitative analysis of 15 senior specialists from primary-care physicians, surgeons, and pediatricians [10]. While time and logistics were important barriers for the surgeons, trust was seen as an important barrier for pediatricians, and patients’ psychosocial and cultural barriers were more important for primary-care physicians. Exploring these wider and divergent perspectives among specialties can facilitate better integration when expanding the story-telling from community medicine to other subject curricula.

The introduction of humanities can prepare medical graduates to deliver person-centered care, help them navigate clinical complexities, and make them capable of uncertainties that are inherent to medicine [11]. Thacker et al. used published graphic medicine (comics, graphic novels, visual narratives) to convey complex situations to explore patients' and doctors' perspectives with a transdisciplinary team approach. The challenge of timetabling and "competition" from "real" medicine makes humanities an "add-on" and creates a narrative that keeps questioning its necessity [11]. This was reflected in many participants' views in our study as well. Moreover, like any other teaching-learning method, story-telling needs to have appropriate assessment methods. Feedback from students as well as teachers, clinical scenarios for the creation of stories and then their peer review, and possible involvement of social science faculty in assessments are various possibilities as far as assessments are concerned. However, like the attitude, ethics, and communication (AETCOM) competencies that have been introduced in the Indian medical graduation, this narrative competency is tricky to assess due to its inherent variability, diversity, and wide scope [12]. However, individual components of story-telling such as writing, narrating, and communication skills can be assessed. A study of standardized patients in orthopedics evaluated skills of information gathering, relationship development, counseling, and communication skills and scored the residents [13]. A jigsaw of imaginary situations in the cascade of care-seeking, medical consultation, and long-term care in select diseases can be used to create stories by the students that can lead to imaginative cooperative learning [14]. Patient ideas, concerns, and expectations introduced in medical education to instill good communication skills have been found to improve the patient-physician narrative gap and have the potential to reduce drug prescriptions [15,16]. The AETCOM in India is a step in this direction, and it intends to improve communication skills where there is an opportunity to introduce the concept of narrative medicine and medical humanities [17].

Story-telling has also been used as a psychotherapy method in children that prepares them for fears and anxieties and improves their analytical and cognitive abilities to facilitate growth and problem-solving capabilities [18]. It has also been used successfully in conveying important health messages like personal hygiene in young children [19].

With the advent of technology, digital story-telling as a tool for better understanding is also becoming very popular. Here, the challenging task of conveying and disseminating complex technical information in the field of medicine is done using multimedia and videos that are used for public viewing, patients, and families as well as for healthcare professionals’ education [20].

Knowledge translation using story-telling in healthcare is also gaining much leverage where it is used to ensure knowledge update in the form of digital multimedia. It has a wide reach and ensures good engagement with patients, caregivers, healthcare professionals, and even policymakers. The introduction of story-telling in medical education, therefore, is very much in tandem with the changing times, where a lot of information consumption is occurring among the stakeholders from health-related websites, documentaries, and YouTube. Role-plays have been traditionally used in community medicine curricula which is an indirect way to learn to make scripts, communicate effectively, have "stage presence," and finally deliver a concept that is important to an audience. A collaborative initiative between Mayo Clinic Centre for Humanities in Medicine and theatre artists is an example of how to learn effective case presentation skills and tell patient stories. Here, students hear the stories from the patients, colleagues, or available as written narratives, identify the crucial elements of this narrative, and share the story creatively interwoven with elements of traditional medical elements [21]. Authors in this initiative rightly point out that such activities have an added possibility of developing skills not just in communication but also a pleasure in telling and eliciting stories that can prevent burnouts that are common in this profession, and this has been demonstrated in other studies also [22]. Lastly, even for a good clinician, story-telling and eliciting skills are important as stated by Willian Osler, taking a good history involves listening to the patient’s story, since often, the patient will tell you the diagnosis. In addition, he rightly said, “The good physician treats the disease; the great physician treats the patient who has the disease” [23], and sensitization in humanities and narrative medicine is useful in this direction.

There are several limitations to our study. Firstly, it was an online survey of feasibility, and voluntary participation in the survey can result in participants with biases self-selecting themselves into the sample. Secondly, the reasons and understanding of the participant with regard to the response cannot be further explored in an online survey. However, since there are not many studies on the feasibility of introducing humanities in India, this study adds value in that direction.

## Conclusions

With ever-advancing medical science, knowledge can become an overwhelming component of learning in medical education. Although the psychomotor domain is unlikely to get affected, there is a possibility of the cognitive domain overriding the affective domain. For the affective domain to develop, we need to cover empathy, altruism, communication, ethics, and humanities, and we can no longer assume that it will happen automatically. A concerted effort toward these can be incorporated into community medicine through story-telling, keeping in mind the challenges related to faculty training, time, and systematic assessment.
